# Virtual reality applications to assist pregnant women: a scoping review

**DOI:** 10.1186/s12884-021-03725-5

**Published:** 2021-03-25

**Authors:** Sadrieh Hajesmaeel-Gohari, Fatemeh Sarpourian, Elaheh Shafiei

**Affiliations:** 1 Medical Informatics Research Center, Institute for Futures Studies in Health, Kerman University of Medical Sciences, Kerman, Iran; 2 Department of Health Information Management, School of Management and Information Sciences, Shiraz University of Medical Sciences, Shiraz, Iran

**Keywords:** Virtual reality, Pregnancy, Delivery, Labor

## Abstract

**Background:**

Virtual reality (VR) is a computer technology that simulates the real world to allow users to communicate with a similar but artificial environment. VR technologies can be used in pregnancy to help mothers gain a better understanding of this significant yet stressful event. The aim of this study was to find and summarize VR applications to help pregnant women during their pregnancy and delivery.

**Methods:**

PubMed, Embase, and Web of Science databases were searched on November 11th, 2020 to access relevant studies. The following data were extracted from the collected studies: first author’s name, year of publication, country, type of study, sample size, study objective, VR components (hardware and software), data gathering method, and study outcomes. Through a descriptive summary and analysis, the results eventually presented.

**Results:**

Nine studies were included in this study. Four studies (44.5%) had used VR technology to reduce the anxiety of pregnant women, four studies (44.5%) had applied VR for decreasing delivery pain, and one study (11%) used VR for exercise trainings. Five studies (56%) used VR headsets and three studies (33.5%) used VR glasses. Most studies showed that VR was a useful method to be used for different purposes in both pregnancy and delivery (*n* = 8, 89%).

**Conclusion:**

The use of VR technology for pregnancy has been increasing in recent years. This technology has different applications in pregnancy, from reducing anxiety and pain to exercise training. However, more studies are required to reach a general common understanding about the efficacy of VR during pregnancy and delivery.

## Background

Virtual reality (VR) is recognized as a computer based technology that simulates the real world. By use of VR, a person can interact with an artificial environment similar to that of reality [1]. Based on the level of presense that VR users experience, VR technology is divided into the three following types: immersive, semi-immersive, and non-immersive. In immersive VR, some features from the real world are added to the virtual setting to induce users’ experience and sense of the virtual environment. In semi-immersive VR, since users are allowed to communicate with the outside environment surrounding them as they are using the technology, users are only partically engaged with the virtural world. Non-immersive VR, however, encompasses computer-generated practices on a desktop and users interact with the virtual environment by means of a device such as a mouse or a joystick [2].

VR technologies are used in different settings for various purposes such as military, sports, education, industry, entertainment, art, and healthcare [3]. In healthcare, VR technology can be used to provide treatment [4], facilitate pain management [5], surgery [6], rehabilitation [7], and medical education [8]. VR can also be used for various purposes in different stages of pregnancy. Embryonic growth and posture can be assessed by means of VR imaging methods [9]. Healthcare providers can also be trained through VR techniques to perform obstetric ultrasonography [10] and laparoscopic surgeries [11].

Most frequently, VR is used to facilitate pregnancy for pregnant women by reducing their anxiety levels and training them to effectively manage their pain during labor [12–14]. A pregnant woman’s blood pressure and heart beat increases during labor and this may intensify if the mother is experiencing pain and anxiety which, as a result, can reduce the amount of blood flow in the uterine. Additionally, anxiety can increase the level of pain during delivery and the possibility of depression after childbirth [15]. Different pharmacological and non-pharmacological methods are used to reduce the pain and anxiety of labor. As a non-pharmacological method, VR technology can provide a simulated environment and distract patients’ concentration on pain signals to something else [16].

VR may be used for other purposes besides the abovementioned ones to assist pregnant women. This study aimed at collecting and investigating the studies that used VR to help pregnant women during their pregnancy and delivery. We formulated our study based on the Population, Concept, and Context (PCC) components. The study reviewed original studies that used VR to help pregnant women (Population) to manage their pregnancy and delivery process in different settings (Context), and finally provided the outcomes of using VR technology (Concept).

## Methods

This was a scoping review study. The Joanna Briggs Institute (JBI) guideline for conducting scoping review studies as the following nine levels was used: defining and aligning the objective and question; developing and aligning the inclusion criteria with the objective and question; describing the planned approach to evidence searching, selection, data extraction, and presentation of the evidence; searching for the evidence; selecting the evidence; extracting the evidence; analysis of the evidence; presentation of the results; summarizing the evidence in relation to the purpose of the review, making conclusions, and noting any implications of the findings were used for conducting this study [17]. The PRISMA extension of scoping reviews (PRISMA-ScR) checklist was used to report results [18].

### Eligibility criteria for selected studies

We searched PubMed, Embase, and Web of Science databases to access relevant studies without any date restriction. The search was done on November 11th, 2020 by S.H. We used (“virtual reality” OR “virtual reality exposure therapy” OR “virtual reality immersion therapy”) AND (pregnancy OR gestation OR obstetric OR delivery OR labor OR labour) search strategy in the field of Title/Abstract to search databases.

We included original interventional and observational studies that used VR for pregnant women to manage their pregnancy and delivery and provided the outcomes. We excluded other types of studies, books, articles written in languages other than English, articles that did not use VR for the purpose of facilitating pregnancy and delivery, articles that used VR to train healthcare providers, articles that used VR for embryonic assessment, and articles whose aim was other than assessing the application of VR.

### Studies review

All retrieved studies were entered in the Endnote reference manager software, and duplicated studies were removed. The remaining studies were assessed based on their titles and abstracts by the two authors (S.H, F.S), separately. The same authors reviewed the full-text of the selected studies from the previous step. Disagreements on study selection were resolved by consensus. Finally, the related studies were selected and included in this research, from which required data was extracted.

### Data extraction

Data were extracted from the included studies using a data extraction form. This form contained the first author’s name, year of publication, country, type of study, sample size, study objective, VR components (hardware and software), data gathering method, and study outcome. Two authors of the present study (S.H, F.S) independently extracted these data from the final list of the included studies.

### Data analysis

Descriptive summary and analysis were used to analyze results. Results were categorized and reported based on general (author’s name, year of publication, country, type of study, sample size, and study objective) and specific characteristics (VR components (hardware and software), data gathering method, and study outcome).

## Results

**Fig. 1:**
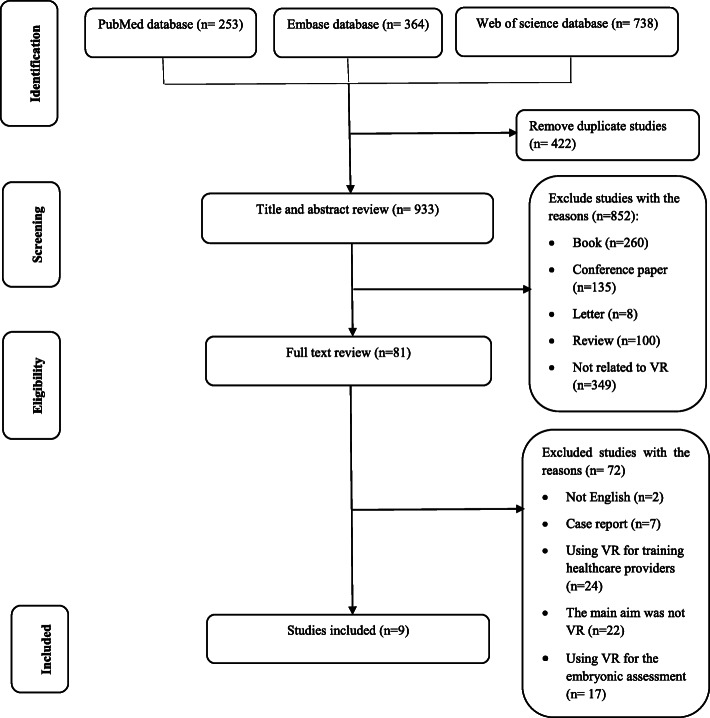
The PRISMA flowchart used in finding relevant studies

From searching PubMed, Embase, and Web of Science databases, 1355 studies were found. After removing 422 duplicate studies, 933 studies remaind whose titles and abstracts were reviewed. Out of the 933 reviewed studies, the full-text of 81 were reviewed for eligibility. Finally, nine studies were included in this research (Fig. 1).

### General characteristics

Most of the studies (*n* = 8, 89%) were published from 2015 to 2020 [13, 14, 19–24]. Only one study was conducted in 2005 [25]. Studies were carried out in different countries. Three studies (33.5%) were performed in the US [13, 19, 20], two in Iran (22%) [23, 24], and the remaining (*n* = 4, 44.5%) in Mexico [21], Netherlands [14], Indonesia [22], and Italy [25]. The majority of the studies (*n* = 5, 56%) were randomized controlled trials [13, 14, 20, 23, 24]. Two (22%) case-control studies [19, 21], and two (22%) before and after studies were included in this research project [22, 25]. A total of 280 pregnant women participated in nine studies. The sample size was from 6 [22] to 97 [14] women, with the majority being 30 [19, 23, 24].

The studies were conducted with the aim of reducing anxiety in pregnant women (*n* = 4, 44.5%) [14, 19, 23, 25], reducing pain (n = 4, 44.5%) [13, 20, 21, 24], and managing exercise training (*n* = 1, 11%) [22]. These studies had considered anxiety in different settings and under different conditions. Sridhar, et al. used VR to reduce anxiety in the first-trimester or the curettage procedure [19]. Noben, et al. provided information about cesarean surgery using VR to reduce the anxiety of pregnant women before cesarean [14]. Jahani, et al. applied VR to reduce the anxiety in primiparous women that required episiotomy repair [23]. Severi, et al. offered virtual fetal touch to improve the interaction between a mother and her fetus to decrease the anxiety she may be feeling [25].

Studies also considered pain in different settings and investgated how pain was managed. Wong, et al. and Frey, et al. used VR to reduce labor pain [13, 20]. Mosso, et al. applied VR to decrease pain and anxiety during cesarean surgery [21]. Jahani, et al. offered VR to manage pain during episiotomy repair [24]. Setiewan, et al. provided exercise trainings by use of VR tools to increase pregnant women’s physical activity [22] (Table 1).

**Table 1:** The general characteristics of the included studies

First author’s name	Year of publication	Country	Type of study	Sample size	Study objective
Sridhar, et al. [19]	2020	US	Case-control	30	Evaluation of the feasibility and effect of VR on anxiety in the first trimester of pregnancy and curettage under local anesthesia
Wong, et al. [20]	2020	US	RCT (Randomized Controlled Trial)	40	Assessing the effect of VR in reducing labor pain
Mosso, et al. [21]	2019	Mexico	Case- control	8	Assessing the effect of VR in reducing labor pain and anxiety in cesarean surgery
Noben, et al. [14]	2019	Netherlands	RCT	97	Assessing the effect of VR in reducing anxiety before cesarean by providing information about the cesarean procedure
Setiawan, et al. [22]	2019	Indonesia	Before-after	6	Development of VR application for pregnant women in order to do sports exercises
Frey, et al. [13]	2018	US	RCT	27	Assessing the effect of VR in reducing labor pain
Jahani, et al. [23]	2016	Iran	RCT	30	Determining the effect of VR on anxiety in primiparous women during episiotomy repair
Jahani, et al. [24]	2015	Iran	RCT	30	Assessing the effect of VR on pain during episiotomy repair
Severi, et al. [25]	2005	Italy	Before-after	12	Anxiety assessment of pregnant mothers using VR (Fetouch system)

### Specific characteristics

Different headsets were used for the VR intervention. Four studies (44.5%) used VR headsets that had to be used with smartphones [13, 14, 19, 22].; yet, only in one study (11%) VR headsets were used on personal computers [21]. Three studies (33.5%) applied glasses in their investigations. Glasses were connected to a DVD player [23, 24] or a personal computer [25] to show VR videos. One study (11%) did not specify the type of used headset [20].

Only three (33.5%) studies used special software to design VR scenarios [14, 22, 25]. Infor-Med, Unity, Google VR, and US3D Touch applications were used in the studies. Other studies (66.5%) applied pre-designed VR videos or did not specify the used software [13, 19–21, 23, 24].

Visual Analogue Scale (VAS) (*n* = 4, 44.5%) [14, 19–21], Numeric Rating Scale (NRS) (*n* = 3, 33.5%) [13, 23, 24] State-Trait Anxiety Inventory (STAI) (*n* = 2, 22%) [23, 25], and Immersive Virtual Environment (IVE) questionnaire (*n* = 1, 11%) [22] were the most commonly used methods for collecting the data, respectively. Also, in three studies, other tools have been used in conjunction with the abovementioned tools [14, 19, 20].

The results of these studies showed that VR is a useful method which can be applied to serve different purposes in pregnancy and upon delivery. Although one study showed anxiety reduction during episiotomy repair, it was not statistically significant [23]. One study also showed that VR was not effective in reducing anxiety before cesarean [14] (Table 2).

**Table 2:** The specific characteristics of the include studies

First author’s name	VR components	Data gathering method	Study outcome
Sridhar, et al. [19]	**Hardware:** Smartphone, VR headset, headphones **Software:** 3D environment (dream beach, Iceland, dolphins)	Pre-procedural anxiety using a modified Amsterdam Preoperative Anxiety and Information Scale (APAIS) and a Visual Analogue Scale (VAS) for anxiety. The semi-structured interviews to assess each participant’s experience with the VR after the procedure	VR was an effective method to reduce anxiety during and after surgery.
Wong, et al. [20]	**Hardware:** VR google headset **Software:** VR blossoming tree, ocean waves, and crackling campfire accompanied by meditative auditory guidance specific to pregnancy and laboring women scenario	Visual Analog Scale (VAS), pre intervention survey, Patient Reported Outcomes Measurement Information System (PROMIS) global health survey, childbirth self-efficacy index (CSEI)	VR was an effective tool for reducing labor pain.
Mosso, et al. [21]	**Hardware:** VR HMD, controller joystick, laptop, **Software:** virtual scenarios (Enchanted Forest, Cliff, and Castle)	Visual Analog Scale (VAS)	The use of VR in cesarean reduced the pain and anxiety of childbirth and was a powerful and safe tool.
Noben, et al. [14]	**Hadware:** VR glasses, Smartphone, tablet **Software:** Infor-Med app. The 360° VR video about all aspects of cesarean delivery	Visual Analogue Scale for Anxiety (VAS-A), The Simulation Sickness Questionnaire (SSQ), Childbirth Perception Scale, Pregnancy and Childbirth Questionnaire, Tilburg Pregnancy Distress Scale	VR was not effective in reducing anxiety before cesarean.
Setiawan, et al. [22]	**Hardware:** Smartphone (Xiaomi Redmi Note 4), VR Shinecon headset, Samsung Gear 2 wearable device. **Software:** Unity, Google VR, Tizen	Immersive Virtual Environment (IVE) questionnaire	VR was a useful teaching exercise tool and measure vital signs with wearable equipment.
Frey, et al. [13]	**Hardware:** Samsung Gear VR HMD, Galaxy S7phone, hand control, head tracking, headphones, S5 phone. **Software:** scene of curious manatees from (www.ocean-rift.com), relaxing music from (www.brain.fm)	Numeric Rating Scale (NRS)	Using the VR approach to gain experience in reducing labor pain and anxiety was useful.
Jahani, et al. [23]	**Hardware:** Glasses (Wrap 920), headphones, stereo sound system with frequency of 60 Hz; resolution 280 × 640 pixels, DVD player/ 3D Blue- Ray Player **Software:** IMAX Dolphin and Whales 1080p Half-SBS AC3	Numeric Rating Scale (NRS), State-Trait Anxiety Inventory (STAI)	VR was a safe and useful non-pharmacological treatment method to reduce episiotomy anxiety and fear of natural childbirth.
Jahani, et al. [24]	**Hardware:** Video player (3D Blu- Ray/ DVD player full HD, model BD660, Indonesia), Video glasses (Wrap920 system, Vuzix factory, US), connection cable, external headphones (stereo 60 Hz, 310 field view), external remote control device, **Software:** 3D film (IMAX Dolphin and Whales 3D 1080p)	Numeric Rating Scale (NRS)	The use of VR was considered a useful tool to reduce pain during episiotomy repair.
Severi, et al. [25]	**Hardware:** Fetouch system, haptic device (phantom, Delta), personal computer, stereo glasses. **Software:** US3D Touch software	State Trait Anxiety Inventory-Form Y (STAI) test	Virtual models reduced the stress of pregnant mothers and lead to better communication between mother and fetus.

## Discussion

This scoping review study was conducted to identify and review the studies that used VR to help pregnant women during pregnancy and delivery. Results showed that VR was used to manage anxiety and pain, and to encourage exercise training during pregnancy and upon delivery.

Pregnancy is one of the most significant and stressful events in women’s lives. Anxiety and fear during pregnancy have adverse medical, mental, biological, and behavioral effects on the mother and her child [26, 27]. One of the main concerns of pregnant women is their delivery process and they fear its pain [28]. On one hand, VR technologies provide information about the operating room and delivery process within an artificial environment similar to that of the real world and help reduce anxiety in pregnant women by allowing mothers to artificially experience childbirth before it actually happens, so mothers are much more ready. On the other hand, VR could be used to decrease women’s focus on her surrounding environment and calm them. One of the other uses of VR in pregnancy is exercise training. Exercise during pregnancy could help to control gestational diabetes, reduce cesarean surgery rates, and ensure good fetal and maternal weight gain [29]. Following an exercise course schedule provided by healthcare centers may be time-consuming and boring for some women. Therefore, it is sufficient to use VR technology instead as it encourages pregnant women to do their exercise at home and at any time they would like [22].

In most of the reviewed studies, VR was proven to be an effective technique in helping pregnant women [13, 19–25]. Only one randomized controlled trial study with 97 participants was shown that the use of VR did not decrease the anxiety that the mothers experienced before their cesarean surgery [14]. Still, this may have been due to the differences in the characteristics of two studied groups, such as the participants’ baseline anxiety scores, level of education, history of psychiatric disorders, and history of an emergency cesarean surgery. A study that was conducted in 2019 also showed that the anxiety levels in women who previously have had emergency cesarean deliveries decreased when VR videos were used [14]. These are some factors that researchers should consider in future studies.

Headsets were used as VR tools in most studies. Similarly, another study showed that Head Mounted Devices (HMD) were frequently used to manage various phobias, and to reduce anxiety and pain [30]. Headsets usually look like boxes that are placed on the head and contain some parts like headphones. In smartphone-based headsets, the phone is placed in the box in order to watch VR videos. Therefore, these headsets may weigh more than virtual reality glasses. A study showed that user discomfort increased when the headset was heavy [31]. This is a critical matter to take into consideration to have successful implementations and increase user satisfaction.

Six studies used pre-designed relaxation videos such as dream beaches, cliffs, dolphins and whales swimming, castles, and forests [13, 19–21, 23, 24]. These natural landscapes can calm pregnant women and distract them from the events taking place around them and as a result reduce their fear and pain. Only three studies designed and developed VR videos by using professional softwares [14, 22, 25]. Noben, et al. developed a VR video that contained all aspects of cesarean delivery to increase the understanding of pregnant women of the event and eventually decrease their anxiety [14]. Setiawan, et al. designed a VR video in which there were trainings to different exercise activities which were useful for pregnant women [22]. Severi, et al. developed a 3D model of the fetus from ultrasound images for VR haptic and visual contact of the mother with her fetus [25]. Although it may be more economical in terms of cost and time to use pre-designed videos, it is more efficient to design videos that match the purpose of study.

VAS, NRS, and STAI were mostly used as tools to determine the amount of pain and anxiety experienced by participants. Another study showed that VAS and NRS were also mostly used to assess endometriosis pain [32]. The VAS is a horizontal line with two endpoints: “no pain” and “worst pain”. Patients mark their pain levels on the 10 cm line from 0 to 100. The NRS is a version of the VAS with a sequential numeric scale from 0 to 10. Patients show their pain level from 11 possible answers; therefore, this scale reflects the intensity of the pain better than VAS [32, 33]. The STAI consists of 40 questions with a 4-point Likert scale. This tool measures state and trait anxiety that represent anxiety about an event and anxiety as an individual characteristic, respectively [34]. Only Setiawan, et al. used the IVE questionnaire as a specific tool to evaluate VR exercise trainings in their study [22]. The IVE questionnaire measures the user experience with 68 items in 9 sections: presence (9 items); engagement (3 items); immersion (5 items); flow (10 items); emotion (11 items); skill (6 items); judgment (9 items); experience consequence (8 items); technology adoption (7 items) [35]. Examining users’ opinions using special questionnaires in the field of VR may be helpful in identifying possible problems and resolving them to ensure a successful implementation of this technology.

Most of the studies were conducted in the US [13, 19, 20] and Iran [23, 24]. Although developed countries such as China and the US have the largest share of VR tools and augmented reality market [36], the results of our study showed that developing countries have also begun to use this technology frequently in recent years. This result indicates that VR has gradually shifted from being a luxury and expensive technology to becoming a more beneficial technology that many people can use worldwide.

Following guidelines can ensure a more successful use of VR technology. Recently, a guideline has been published that takes VR clinical trial studies into consideration, especially in healthcare. This guideline provides advice in three stages of clinical trial study designs. The first stage focuses on user-centered designs of VR content, the second stage focuss on the initial evaluation of the acceptability, user satisfaction, and clinical efficiency of the used VR technology; and finally, the third stage focuses on conducting randomized controlled trial studies to assess the efficacy of VR in different groups [37].

Based on the researchers’ knowledge, this was the first study that investigated the application of VR in assisting pregnant mothers. As for the limitations of this study, the study protocol was not written or registered. Also, keywords were searched only in the Titles/Abstract field of the three databases, which may have excluded some other relevant studies.

## Conclusion

VR technology has different applications in pregnancy, from reducing anxiety and pain to exercise training. VR technologies can decrease the anxiety of pregnant women and their pain of delivery by informing the mothers about the operating room and delivery process in an artificial setting before women encounter the situation in reality. Also, VR helped to distract women from the events taking place around them which inturn helped to decrease their stress and anxiety. Although studies have shown that VR is an effective method in helping pregnant women, it is essential to take the available guidelines into consideration to ensure a successful implementation of this technology in the future.

## Data Availability

Not applicable.

## Abbreviations

VR: Virtual Reality
PCC: Population, Concept, and Context
JBI: Joanna Briggs Institute
PRISMA-ScR: The PRISMA extension of scoping reviews
VAS: Visual Analogue Scale
NRS: Numeric Rating Scale
STAI: State-Trait Anxiety Inventory
IVE: Immersive Virtual Environment
RCT: Randomized Controlled Trial
US: United States
